# Validity of self-reported weight, height, and body mass index among university students in Thailand: Implications for population studies of obesity in developing countries

**DOI:** 10.1186/1478-7954-7-15

**Published:** 2009-09-25

**Authors:** Lynette LY Lim, Sam-ang Seubsman, Adrian Sleigh

**Affiliations:** 1 National Centre for Epidemiology and Population Health, Australian National University, Canberra, Australia; 2 School of Human Ecology, Sukhothai Thammathirat Open University, Pakkret, Thailand

## Abstract

**Background:**

Large-scale epidemiological studies commonly use self-reported weights and heights to determine weight status. Validity of such self-reported data has been assessed primarily in Western populations in developed countries, although its use is widespread in developing countries. We examine the validity of obesity based on self-reported data in an Asian developing country, and derive improved obesity prevalence estimates using the "reduced BMI threshold" method.

**Methods:**

Self-reported and measured heights and weights were obtained from 741 students attending an open university in Thailand (mean age 34 years). Receiver operator characteristic techniques were applied to derive "reduced BMI thresholds."

**Results:**

Height was over-reported by a mean of 1.54 cm (SD 2.23) in men and 1.33 cm (1.84) in women. Weight was under-reported by 0.93 kg (3.47) in men and 0.62 kg (2.14) in women. Sensitivity and specificity for determining obesity (Thai BMI threshold 25 kg/m^2^) using self-reported data were 74.2% and 97.3%, respectively, for men and 71.9% and 100% for women. For men, reducing the BMI threshold to 24.5 kg/m^2 ^increased the estimated obesity prevalence based on self-reports from 29.1% to 33.8% (true prevalence was 36.9%). For women, using a BMI threshold of 24.4 kg/m^2^, the improvement was from 12.0% to 15.9% (true prevalence 16.7%).

**Conclusion:**

Young educated Thais under-report weight and over-report height in ways similar to their counterparts in developed countries. Simple adjustments to BMI thresholds will overcome these reporting biases for estimation of obesity prevalence. Our study suggests that self-reported weights and heights can provide economical and valid measures of weight status in high school-educated populations in developing countries.

## Introduction

Body mass index (BMI), calculated as weight in kilograms divided by the square of height in meters, is widely used to classify body size as underweight, normal, overweight, or obese. In Western populations, an adult with BMI of 30 kg/m^2 ^or over is considered obese [1]. Epidemiological studies involving large numbers of people frequently replace actual weight and height measurements with self-reported data. Many studies have shown that self-reports correlate highly with measured data [2-4]. But studies also consistently noted systematic bias in self-reported data, with height generally overestimated and weight generally underestimated [5]. Thus, self-reported BMI is most often lower than measured BMI [5,6]; as a result, some obese individuals are misclassified as nonobese, leading to underestimation of obesity prevalence.

Dauphinot (2008) proposed a simple and intuitive method to obtain better estimates of obesity prevalence from self-reports [7]. Instead of defining obesity at the 30 kg/m^2 ^threshold, the method proposes a "reduced BMI threshold" derived using receiver operator characteristic (ROC) techniques. In their population, by reducing the BMI threshold to 29.2 kg/m^2^, they obtained obesity prevalence estimates from self-reports that were not significantly different from the true values. For application in populations with different characteristics, it was recommended that reduced BMI thresholds be computed from ancillary data collected on a limited representative sample [7].

Research on the validity of self-reported weight, height, and BMI have been performed primarily on Western populations in developed countries. A recent systematic review [5] of the validity of self-reported BMI comprising 64 studies found only two conducted on Asian populations, both Japanese [4,8]. The accuracy of self-reported BMI was found to vary by age, sex, socioeconomic status, actual weight status, ethnicity, and perceived body image [4,9-12]. The accuracy of self-reported weight and height in Asian people may differ from Western populations because of differences in body size and cultural norms. For example, an international comparison of 22 countries found that the perceptions of overweight and attempts to lose weight were highest in Asian nations [13]. Findings on the validity of self-reported anthropometric measurements in Western populations may not generalize to Asian populations.

The present work was motivated by the need to assess weight status in a large cohort study of health risk transition in Thailand, the Thai Cohort Study (TCS) [14]. More than 80,000 students from across Thailand enrolled at the Sukhothai Thammathirat Open University (STOU) participated in the baseline TCS survey, reporting their weight and height as part of a 20-page mailed-out questionnaire. Here, we investigate the validity of self-reported cohort weights and heights using ancillary data from an independent sample of 741 STOU men and women. We assess the validity of body size estimates based on these self-reported weights and heights in a Thai population, and we evaluate obesity prevalence estimates using the "reduced BMI threshold" method.

## Methods

### Study population

STOU students attend an on-campus course in their final semester devoted to professional and ethical issues. Each batch is about 350 students from all over the country, usually well-balanced between male and female. These students were recruited for this study on three occasions between December 2005 and May 2007. Participation was voluntary. Students were encouraged to participate as a "contribution to society" and were asked to self-report their weight and height by filling in a one-page form. They were not informed at the time about actual measurements. They were given a "BMI kit" that explained the utility and computation of BMI when they returned the self-report form and were invited at that time to volunteer for measurements by our research assistant.

### Data collection

The self-report form asked for height (without shoes) in centimeters and weight in kilograms to the nearest whole centimeter and kilogram, respectively. The self-report and measured data were obtained on the same day. All of the weight and height measurements were performed by one research assistant on each occasion. Weight was measured using an electronic Seca scale, which was calibrated and checked for accuracy by the company representing the manufacturer the day before it was used. Height was measured to the nearest half-centimeter using a stadiometer. The students were instructed to stand with their feet together and to look straight ahead.

### Height and weight variables

Measured BMI was defined as the BMI calculated from measured weight and height, and self-reported BMI as the BMI calculated from self-reported weight and height. Discrepancies were assessed by the differences between self-reported and measured weight, height, and BMI. Weight discrepancies of more than 5 kg, height discrepancies of more than 10 cm, and BMI below 10 kg/m^2 ^or above 45 kg/m^2 ^were checked for data entry errors.

Obesity was defined as BMI≥25 kg/m^2 ^and overweight as BMI≥23 kg/m^2^, in accordance with WHO criteria for Asian populations [15]. The sensitivities and specificities of the obese and overweight categories based on self-reported BMI were calculated using measured BMI as the standard of comparison: sensitivity is the probability of true positives and specificity the probability of true negatives. Positive predictive values (PPV) and negative predictive values (NPV) were also computed. PPV (for obesity) reflects the probability that a person classified as obese from self-reported information is truly obese, and is the most important indicator of the utility of self-reported weights and heights as a tool for determining obesity. NPV (for obesity) reflects the probability that a person not classified as obese is truly not obese. PPV and NPV depend on sensitivity and specificity as well as on prevalence.

### Statistical methods

As the validity of self-reported weight and height differ between men and women, separate analyses were done for each sex. Statistical analyses were performed using Stata Version 9.0. Statistical methods included the use of descriptive parameters (mean, standard deviation), Spearman's rank correlation coefficient, chi-squared and paired t-test. Confidence intervals were calculated using the normal approximation for standard errors of proportions. Binary variables measuring rounding effect were also computed for self-reported weight and height - "end-digit preference" for values ending in zero or five were compared to the other values ending in 1 to 4 or 6 to 9 [2,15]. The proportion with end-digit preference was compared to the expected proportion of 20%.

### Reduced BMI threshold

The Dauphinot method [7] obtains improved estimates of prevalence from self-reported weights and heights by lowering the threshold used to define obesity (or overweight/obese). The reduced BMI threshold defining obesity (or overweight/obese) is determined from receiver operator characteristic (ROC) curve analysis by selecting the (highest) BMI value, which maximizes the percentage of people correctly classified.

## Results

The subjects were aged 21 to 62 years. The mean ages for men and women were 34.7 years (SD 8.4) and 34.1 years (SD 7.9), respectively.

There were strong correlations between measured and self-reported values in weight, height, and BMI for both men and women (Spearman's correlation for men and women respectively: 0.95 and 0.97 for weight, 0.94 and 0.94 for height, 0.91 and 0.95 for BMI) (Table 1).

**Table 1:** Self-reported and measured anthropometric measurements, their discrepancies and correlations separately for men (n = 358) and women (n = 383)

	**Self-reported** **Mean (SD)**		**Measured** **Mean (SD)**		**Discrepancy** **(Self-reported - Measured)** **Mean (95% CI)**		**Correlation** **ρ (p-value)**	
**Weight**, kg								
Men	67.6	(11.7)	68.5	(12.1)	-0.93	(-1.29, -0.57)***	0.95	(< 0.0001)
Women	53.8	(9.1)	54.5	(9.8)	-0.62	(-0.83, -0.40)***	0.97	(< 0.0001)
								
**Height**, cm								
Men	168.6	(6.3)	167.1	(6.0)	1.54	(1.31, 1.77)***	0.94	(< 0.0001)
Women	157.9	(5.4)	156.6	(5.2)	1.33	(1.15, 1.51)***	0.94	(< 0.0001)
								
**BM**I, kg/m^2^								
Men	23.7	(3.6)	24.5	(3.8)	-0.77	(-0.92, -0.62)***	0.91	(< 0.0001)
Women	21.6	(3.3)	22.2	(3.7)	-0.62	(-0.72, -0.51)***	0.95	(< 0.0001)

***p < 0.0001 (p-values from paired t-tests)

Both men and women statistically significantly under-reported their weights and over-reported their heights (Table 1). On average, men under-reported weight by 0.93 kg and over-reported height by 1.54 cm. Women under-reported weight by 0.62 kg and over-reported height by 1.33 cm. Consequently, BMI calculated from self-reported data significantly under-reported measured BMI by 0.77 kg/m^2 ^and 0.62 kg/m^2 ^for men and women, respectively.

### Reporting discrepancies by measured BMI category

Table 2 examines the effect of actual weight status on discrepancies in weight and height reporting. Increasing weight status was strongly associated with more pronounced under-reporting of weight as well as over-reporting of height for both sexes. On average, weight was under-reported by 0.3 kg, 1.2 kg, and 1.8 kg, respectively, in normal weight, overweight, and obese individuals. Underweight individuals tended to over-report weight. Height was over-reported in both sexes. In normal weight individuals, the over-reporting of height, by 1.18 cm in men and 1.13 cm in women , was highly statistically significant. In overweight men and women, height was significantly over-reported by 1.69 cm and 1.36 cm, respectively, and in obese men and women, by 1.92 cm and 1.83 cm, respectively. Underweight females over-reported height by 1.7 cm, but underweight males under-reported height by 1.4 cm.

**Table 2:** Discrepancies in self-reported weights and heights and end-digit rounding preference by measured BMI category separately for men and women

			**Weight**				**Height**			
			
**Measured BMI Category^a^**	**N (%)**		**Discrepancy (SR^b ^- Measured)** **Kg, mean (95%CI)**		**Percent reporting end-digit 0 or 5** **% (95% CI)**		**Discrepancy (SR^b ^- Measured)** **cm, mean (95%CI)**		**Percent reporting end-digit 0 or 5** **% (95% CI)**	
**Men**										
Underweight (<18.5)	5	(1)	1.13	(0.40, 1.86)*	0%	(0%, 52%)	-1.40	(-5.78, 2.98)	0%	(0%, 52%)
Normal (18.5-<23)	133	(37)	-0.08	(-0.46, 0.29)	29%	(21%, 37%)*	1.18	(0.80, 1.56)***	32%	(24%, 41%)***
Overweight (23-<25)	88	(25)	-1.34	(-2.18, -0.50)**	36%	(26%, 47%)***	1.69	(1.20, 2.17)***	36%	(26%, 47%)***
Obese (25+)	132	(37)	-1.58	(-2.27, -0.89)***	36%	(28%, 45%)***	1.92	(1.57, 2.27)***	43%	(35%, 52%)***
										
**Women**										
Underweight (<18.5)	36	(9)	1.18	(0.30, 2.06)**	19%	(8%, 36%)	1.65	(0.79, 2.51)***	44%	(28%, 62%)***
Normal (18.5-<23)	229	(60)	-0.38	(-0.59, -0.18)***	23%	(17%, 29%)	1.13	(0.93, 1.34)***	29%	(23%, 35%)***
Overweight (23-<25)	54	(14)	-0.94	(-1.45, -0.42)***	33%	(21%, 47%)*	1.36	(0.86, 1.87)***	39%	(26%, 53%)***
Obese (25+)	64	(17)	-2.21	(-2.89, -1.53)***	33%	(22%, 46%)*	1.83	(1.30, 2.35)***	41%	(29%, 54%)***

^a ^Using Thai classification (15)

^b ^SR = self-reported

*p < 0.05; **p < 0.01; ***p < 0.001 (for discrepancies, p-values were from paired t-tests; for end-digit tests, p-values were from exact binomial tests of proportion = 0.20)

Overall, 35% of men and 28% of women showed "end-digit preference" (the tendency to round digits to zero or five) in reporting weight, and 37% and 34%, respectively, in reporting height. Except for underweight men, for which there were too few subjects, and underweight and normal weight women reporting weight, all other subgroups showed end-digit preference that was significantly different from 20% (Table 2). End-digit preference appeared to be a little more pronounced with increasing weight status.

### Diagnostic test values

Table 3 shows the test values when self-reported weight and height were used as tools to determine weight status (obesity and overweight/obese). Defining obesity as BMI>25 kg/m^2^, specificity was very high for both men and women (97.2% and 100%, respectively), as were positive predictive values (94.2% and 100%). Sensitivity was considerably lower, 74.2% and 71.9%, respectively, for men and women. Negative predictive values were 86.6% and 94.7% for men and women, respectively.

**Table 3:** Test values for diagnosis of obesity and overweight/obesity based on self-reported data comparing standard Thai BMI thresholds and reduced BMI thresholds, and effects on prevalence estimates

	**Sensitivity**	**Specificity**	**Positive predictive value (PPV)**	**Negative predictive value (NPV)**	**Measured prevalence**	**Self-reported prevalence**	**TCS^a ^self-reported prevalence**
*Obesity*							
**Men**							
Thai BMI threshold (≥25)	74.2	97.3	94.2	86.6	36.9	29.1	22.7
Reduced BMI threshold (≥24.5)	81.1	93.8	88.4	89.5	-	33.8	28.0
							
**Women**							
Thai BMI threshold (≥25)	71.9	100.0	100.0	94.7	16.7	12.0	10.0
Reduced BMI threshold (≥24.4)	85.9	98.1	90.1	81.4	-	15.9	12.2
							
*Overweight/Obese*							
**Men**							
Thai BMI threshold (≥23)	77.7	93.5	95.0	72.5	61.5	50.3	44.4
Reduced BMI threshold (≥22.4)	87.7	86.2	91.0	81.4	-	58.9	51.8
							
**Women**							
Thai BMI threshold (≥23)	77.1	99.2	97.8	90.7	30.8	24.3	19.6
Reduced BMI threshold (≥22.5)	87.3	97.0	92.8	94.5	-	29.0	23.2

^a ^Thai Cohort Study (n = 38,815 men, 47,070 women)

When self-reports were used to determine overweight/obese in both sexes, sensitivity was higher and specificity lower than when used to determine obesity. Specificity was 94% and 99%, and sensitivity was 78% and 77% for men and women, respectively. PPVs were similar in men, but decreased slightly in women. NPVs decreased by much larger amounts -- 12 percentage points in men and four in women.

### Prevalence estimates

Obesity prevalences based on self-reported data for men and women were 29.1% and 12.0%, respectively. These prevalences, underestimated by 7.8% in men and 4.7% in women, were statistically significantly lower than the "true" prevalences based on measured values of 36.9% and 16.7% (Table 3).

When ROC techniques as recommended by Dauphinot were applied, the proportions correctly classified as obese were maximized at BMI thresholds of 24.5 kg/m^2 ^for men and 24.4 kg/m^2 ^for women. Using these reduced thresholds, obesity prevalences increased to 33.8% for men and 15.9% for women, which were not statistically significantly different from the "true" prevalences (Table 3). Stated another way, application of the "reduced BMI threshold" method significantly reduced under-estimation of obesity prevalence estimates to 3.1% in men and 1.1% in women.

For overweight/obese, the reduced BMI thresholds were 22.4 kg/m^2 ^for men and 22.5 kg/m^2 ^for women (Table 3). These thresholds increased the prevalence estimates for men from 50.3% to 58.9%, which was not statistically significantly different from the "true" prevalence of 61.5%. For women, the prevalence estimate increased from 24.3% to 29.0%, also not significantly different from the "true" prevalence of 30.8%.

By maximizing the proportion of individuals correctly classified, the Dauphinot method increases sensitivity, decreases specificity, and decreases the PPV [7]. In this sample, PPVs were decreased by between 4% and 10% (Table 3).

When the reduced BMI thresholds were applied to the entire TCS sample, obesity prevalence estimates increased by 5.3% (from 22.7% to 28.0%) for men and by 2.2% (10.0% to 12.2%) for women (Table 3). Prevalence estimates of overweight/obese increased by 7.4% for men and 3.6% for women (Table 3).

## Discussion

This study examined the validity of self-reported weights and heights for determining obesity and overweight/obese in Thai men and women. Previous studies examining this issue have been on populations in developed countries and, except for two studies in Japan, none were conducted in Asian countries. Understanding the validity and accuracy of self-reported anthropometric data is important so that such data can be used with confidence, or at least with knowledge of its limitations, when economizing on resources is necessary for accomplishing the research. To the best of our knowledge, our study is the first to examine this topic in a developing nation in Asia. In many rapidly urbanizing countries such as Thailand, the emerging obesity epidemic is a growing public health problem requiring urgent large-scale community intervention [16]. Self-reported weights and heights in these settings would provide a practical and economical means of monitoring the effectiveness of intervention program trends.

Our study showed results consistent with findings in Western populations. Previous studies have reported very high correlations, more than 0.90, between self-reported and measured weight, height, and BMI in men and women under 60 years of age [2-4,17]. In our study, correlations were high in both sexes, ranging from 0.91 to 0.95. In most studies, as well as in ours, correlations for height were marginally lower than for weight. This phenomenon could be a reflection of many individuals' greater awareness of their weight than of their height.

Most studies found an under-reporting of weight and an over-reporting of height. Studies of general population samples in France [2], Scotland [18], the US [6,11], the UK [3], Brazil [17], Australia [19], Sweden [20], Canada [21], and Italy [22] reported weight discrepancies ranging from -0.54 kg to -0.76 kg for men and from -0.85 kg to -2.5 kg for women. The men in our Thai sample showed weight and height discrepancies that were comparable to these. For the women, discrepancy in mean height was comparable, but the discrepancy of mean weight, at -0.62 kg, was smaller.

Our results were more similar to those from Western countries than to Japan. The Japanese study showed extremely small weight and height discrepancies -- less than 0.05 kg for weight and less than 0.08 cm for height in both sexes. The high degree of accuracy was attributed partly to the practice of annual health checkups in Japan and to the sample being a homogenous group of middle-class public servants recruited from a single workplace.

Actual body weight status had been found to be a major determinant of weight under-reporting in many studies [2,4,18-21]. A similar pattern was seen in our study, where weight reporting discrepancy was not significant for normal weight individuals, but highly significant (about 2 kg) for obese men and women.

The influence of weight status on height reporting was less consistent. The Japanese study [4] found no increase in discrepancy. The French [2], Australian [19], and Swedish [20] studies found moderate increases. The Canadian study [21] found significant trends only in men, while the Scottish study [18] showed a trend in the opposite direction. Our study showed moderate increases in height reporting discrepancies in both sexes. The finding in our study of significant height over-reporting among individuals of normal weight status was not observed in other studies. This may be because short stature is socially undesirable in Thai culture, resulting in a tendency to over-estimate height. But another explanation is that Thai identity cards show head-and-shoulder photos against a wall scale, and heights are recorded at the top of the hair line. In our stadiometer measurements, height was read against the top of the skull, ignoring hair thickness. This could account for most of the difference between adult Thai self-reports of height and actual measurements using the criteria we adopted.

The proportions of people with end-digit preference increased slightly with weight status, suggesting that the greater reporting discrepancy among overweight and obese individuals may be due to a greater tendency to round weight down and round height up.

Specificity for detecting obesity in our study, as in other studies [4,17,19-21], was very high, typically between 97% and 100%. This indicated that few individuals reported weight and height that would put them in the obese category unless they really were obese. Sensitivity was more variable, with values ranging from 59% to 89% [4,17,19-21]. Sensitivity values in our study (74.2% and 71.9% for men and women, respectively) were lower than in the Japanese [4] and Scottish [18] studies, higher than in the Canadian [21] study, and comparable to the Swedish [20], Australian [19], US [10], and Brazilian [17] studies.

Compared to obesity determination, when self-reported weights and heights were used to determine overweight/obese, specificity was generally lower and sensitivity higher. This suggests that, as a tool for determining weight status, self-reported weights and heights give more accurate assessment of obesity than of overweight/obese.

Previous research has been consistent in finding PPVs and NPVs for obesity that were higher in women than in men. This was also the case in our study, where PPV and NPV for obesity were 100% and 95%, respectively, in women, and 94% and 87% in men. This suggests that the determination of weight status from self-reported weights and heights was more accurate in women than men, a point noted in a few previous studies [2,22,23].

Overall, obesity prevalence underestimation in our study was similar to many studies (e.g., Canada [21], Sweden [20], and Australia [19]), but higher than in others (e.g., Switzerland [7] and Japan [4]). Application of the "reduced BMI threshold" method significantly reduced under-estimation of obesity prevalence estimates and decreased PPVs in our study. The decrease in PPVs implies that reduced thresholds should not be used for individual weight status classification for clinical or diagnostic purposes, which supports Dauphinot's [7] observation that "... the revised obesity threshold should be applied only on population data."

Several limitations and observations should be noted.

Although our study suggests self-reported weights and heights can be used successfully to determine weight status, we caution against extrapolating this finding to Thailand as a whole because our participants were sampled from STOU, which requires completion of high school education for enrollment. In contrast, less than half of the Thai general population has completed high school [24]. Another difference is in the age distribution. Being an open university, STOU admits students of any age, but the majority is under 40 years of age (87% of TCS members are under 40 years old). The use of volunteers, who may be more willing to honestly report their weights and heights than the general population, may also influence the findings in this study. Further, the bias in self-reports obtained on campus, as in the present study, may differ from self-reports obtained in the home setting, where respondents may have access to weight and height instruments.

Prevalences of obesity were higher in the ancillary study than in the TCS (29% versus 23% for men; 12% versus 10% for women). This could be due to the lower average age of the TCS sample compared to the ancillary sample, by 2.5 years for men and 5.1 years for women.

An exceptionally high proportion (21%) of women in the TCS sample were underweight (BMI <18.5 kg/m^2^) based on self-reported data [25]. The underweight women in the ancillary study tended to *over*-report their weight, a finding consistent with the behavior of underweight women in other studies (e.g., Danubio [22]). This suggests that the prevalence of underweight women in TCS may be even *higher *than the one-fifth of the female population indicated from self-reported data. But due to the small numbers (only 36 women in the ancillary study (9%) were underweight), further data are needed to examine the validity of self-reported data among underweight women.

In summary, educated young Thais under-report weight and over-report height in ways similar to their counterparts in developed countries. These reporting biases can be overcome with a simple adjustment of BMI thresholds to provide obesity prevalence estimates within statistical error of the true prevalence. Our findings suggest that self-reporting of weights and heights can provide economical and valid measures of weight status in high school-educated populations in developing countries, but further work is needed to confirm these findings in other settings.

## Competing interests

The authors declare that they have no competing interests.

## Authors' contributions

LL, SS, and AS jointly conceived the study. LL performed the analyses and drafted the manuscript. SS managed and coordinated the data collection. AS participated in the study conduct and manuscript preparation. All authors read and approved the final manuscript.

## References

[B1] WHO (2000). Obesity: preventing and managing the global epidemic.

[B2] Niedhammer I, Bugel I, Bonenfant S, Goldberg M, Leclerc A (2000). Validity of self-reported weight and height in the French GAZEL cohort. Int J Obes Relat Metab Disord.

[B3] Spencer EA, Appleby PN, Davey GK, Key TJ (2002). Validity of self-reported height and weight in 4808 EPIC-Oxford participants. Public Health Nutr.

[B4] Wada K, Tamakoshi K, Tsunekawa T, Otsuka R, Zhang H, Murata C, Nagasawa N, Matsushita K, Sugiura K, Yatsuya H, Toyoshima H (2005). Validity of self-reported height and weight in a Japanese workplace population. Int J Obes (Lond).

[B5] Gorber SC, Tremblay M, Moher D, Gorber B (2007). A comparison of direct vs. self-report measures for assessing height, weight and body mass index: a systematic review. Obes Rev.

[B6] McAdams MA, Van Dam RM, Hu FB (2007). Comparison of self-reported and measured BMI as correlates of disease markers in US adults. Obesity (Silver Spring).

[B7] Dauphinot V, Wolff H, Naudin F, Gueguen R, Sermet C, Gaspoz JM, Kossovsky MP (2009). New obesity body mass index threshold for self-reported data. J Epidemiol Community Health.

[B8] Nakamura K, Hoshino Y, Kodama K, Yamamoto M (1999). Reliability of self-reported body height and weight of adult Japanese women. J Biosoc Sci.

[B9] Bostrom G, Diderichsen F (1997). Socioeconomic differentials in misclassification of height, weight and body mass index based on questionnaire data. Int J Epidemiol.

[B10] Gillum RF, Sempos CT (2005). Ethnic variation in validity of classification of overweight and obesity using self-reported weight and height in American women and men: the Third National Health and Nutrition Examination Survey. Nutr J.

[B11] Kuczmarski MF, Kuczmarski RJ, Najjar M (2001). Effects of age on validity of self-reported height, weight, and body mass index: findings from the Third National Health and Nutrition Examination Survey, 1988-1994. J Am Diet Assoc.

[B12] Paeratakul S, White MA, Williamson DA, Ryan DH, Bray GA (2002). Sex, race/ethnicity, socioeconomic status, and BMI in relation to self-perception of overweight. Obes Res.

[B13] Wardle J, Haase AM, Steptoe A (2006). Body image and weight control in young adults: international comparisons in university students from 22 countries. Int J Obes (Lond).

[B14] Sleigh AC, Seubsman SA, Bain C (2008). Cohort profile: The Thai Cohort of 87,134 Open University students. Int J Epidemiol.

[B15] taskforce Io, WHO (Western Pacific Region) (2000). The Asia-Pacific perspective: redefining obesity and its treatment. International Associates for the study of obesity.

[B16] Misra A, Khurana L (2008). Obesity and the metabolic syndrome in developing countries. J Clin Endocrinol Metab.

[B17] Fonseca MJ, Faerstein E, Chor D, Lopes CS (2004). [Validity of self-reported weight and height and the body mass index within the "Pro-saude" study]. Rev Saude Publica.

[B18] Bolton-Smith C, Woodward M, Tunstall-Pedoe H, Morrison C (2000). Accuracy of the estimated prevalence of obesity from self reported height and weight in an adult Scottish population. J Epidemiol Community Health.

[B19] Taylor AW, Dal Grande E, Gill TK, Chittleborough CR, Wilson DH, Adams RJ, Grant JF, Phillips P, Appleton S, Ruffin RE (2006). How valid are self-reported height and weight? A comparison between CATI self-report and clinic measurements using a large cohort study. Aust N Z J Public Health.

[B20] Nyholm M, Gullberg B, Merlo J, Lundqvist-Persson C, Rastam L, Lindblad U (2007). The validity of obesity based on self-reported weight and height: Implications for population studies. Obesity (Silver Spring).

[B21] Shields M, Gorber SC, Tremblay MS (2008). Estimates of obesity based on self-report versus direct measures. Health Rep.

[B22] Danubio ME, Miranda G, Vinciguerra MG, Vecchi E, Rufo F (2008). Comparison of self-reported and measured height and weight: implications for obesity research among young adults. Econ Hum Biol.

[B23] Flood V, Webb K, Lazarus R, Pang G (2000). Use of self-report to monitor overweight and obesity in populations: some issues for consideration. Aust N Z J Public Health.

[B24] Wittayakorn C (2006). 2004-2005 Thai youth education status report: the root cause and guidelines for solutions.

[B25] Banwell C, Lim L, Seubsman SA, Bain C, Dixon J, Sleigh A (2009). Body mass index and health-related behaviours in a national cohort of 87,134 Thai open university students. J Epidemiol Community Health.

